# Waging Peace through Neglected Tropical Disease Control: A US Foreign Policy for the Bottom Billion

**DOI:** 10.1371/journal.pntd.0000346

**Published:** 2009-01-27

**Authors:** Peter J. Hotez, Tommy G. Thompson

**Affiliations:** 1 Global Network for Neglected Tropical Diseases and Sabin Vaccine Institute, Washington, D.C., United States of America; 2 Department of Microbiology, Immunology, and Tropical Medicine, The George Washington University, Washington, D.C., United States of America

*“What better way to knock down…the barriers of ethnic and religious groups that are afraid of America…than to offer good medical policy and good health to these countries?”—Tommy G. Thompson, 2004* [*29*]

It comes as no surprise that poverty breeds political instability, and some of the most troubled regions on our planet are disproportionately represented by nations with large numbers of people who live on less than US$1 per day. It has been observed that most of the people living in the societies of the “bottom billion,” i.e., the world's poorest people who live on little or no money, are either currently engaged in a civil war or have recently been through one, and today civil war breaks out almost exclusively in low-income countries [1]. In such areas the risk of conflict has been shown to increase as the starting income of the country diminishes [1].

With respect to international conflicts and civil war, poor infant and child health represent important fellow travelers with poverty. Previous analyses by one of us show that nations with the highest infant and under-five child mortality rates are the ones most likely to engage in war [2]–[4]. Childhood infections together with malnutrition by far account for the greatest burden of pediatric morbidity and mortality in developing countries, so that infectious diseases operate in synergy with poverty to promote conflict. Among the possible factors accounting for the relationship between infection and conflict are the adverse impact of the former on the agricultural workforce, agricultural productivity, the health of families, and the stability of communities and community leaders [5]–[7].

The neglected tropical diseases (NTDs) represent possibly the worst of all diseases in terms of their destabilizing effects and their relationship to conflict. The NTDs are the most common infections of the bottom billion, in whom they cause chronic, debilitating, disabling, and disfiguring effects [8]. One of the most important features of the NTDs is their tendency not only to occur in the setting of poverty, but also to exacerbate poverty and to destabilize communities [8] (Box 1). For example, the chronic and irreversible limb lymphedema caused by lymphatic filariasis reduces agricultural productivity by causing people to alter their activities or even stop working altogether [9]. Blinding eye disease caused by trachoma and onchocerciasis (river blindness) can also disable subsistence agricultural workers [8], as do hookworm infection and schistosomiasis because they each produce severe anemia, which weakens both the mind and the body [10],[11]. In developing countries, hookworm and schistosomiasis frequently occur together and are co-endemic with malaria to produce profound anemia [12]–[14]. Moreover, when NTDs such as river blindness become pervasive, subsistence farmers are sometimes forced to flee or abandon their fields and migrate to areas with poor soils or inadequate climate [15]. For all of these reasons, NTDs have a pivotal role in the world's food crisis, particularly in developing countries. In addition to their agricultural impact, the NTDs, especially hookworm and other helminth infections, contribute to the “poverty trap” through their adverse effects on education because they interfere with child cognition and learning [16]. Through such mechanisms, chronic hookworm infection in childhood reduces future wage earning by 43% [17]. Worms also injure pregnant women and cause low birth weight [10],[11].

The adverse impact of NTDs on agricultural productivity, education, future wage earning, and the health of mothers and children in low-income countries accounts for the observation that there are multiple and intimate connections between pervasive NTDs and conflict [18]–[21]. Beyrer et al. have identified important links between NTDs and war, conflict, and human rights, especially from lymphatic filariasis and other NTDs among marginalized ethnic populations in Myanmar and vector-borne NTDs such as leishmaniasis and Chagas disease that emerge with civil conflicts and guerilla activities in Colombia and southern Mexico [20]. The war-torn belt in Africa that stretches from the East in Sudan, through Central African Republic and the Democratic Republic of the Congo in Central Africa, to Angola in West Africa exhibits some of the highest rates of NTDs in the world, including the important vector-borne NTDs human African trypanosomiasis (HAT) and leishmaniasis [19],[21]. Tens of millions of people in this region are afflicted with hookworm and other soil-transmitted helminth infections, schistosomiasis, and concurrent co-infections with hookworm and schistosomiasis [14].

Shown in Table 1 is a list of selected countries that comprise diplomatic “hot spots” for the United States of America, in terms of their volatility and current or recent track record of conflict, or because of their deteriorated relationships with the US Government [15], [19], [20], [22]–[27]. All of these nations exhibit high rates of NTDs, with up to 50% of the populations living in conflicted areas suffering from one or more NTD. In sub-Saharan Africa, hookworm infection and schistosomiasis are two of the most prevalent NTDs [14], and are also co-endemic with lymphatic filariasis, onchocerciasis, trachoma, and HAT [24]. Moreover, while many of the areas in conflict are low-income countries, several of them, especially in the Middle East, are middle-income countries and yet still exhibit high NTD prevalence rates, especially with leishmaniasis [27]. Many of these countries also belong to the Organization of Islamic States [4]. Overall, the NTDs are the most common clinical conditions in areas of human conflict and instability, and account for a huge burden of not only disease and disability, but also poverty and disruption of civil society.

**Table 1:** The NTDs in Selected US Diplomatic “Hot Spots”: Conflict and Post-Conflict Countries, and Countries with Unstable Governments or Strained Relationships with the US Government.

Country	Population	Prevalence of NTDs						
		Ascariasis	Trichuriasis	Hookworm	Schistosomiasis	Lymphatic Filariasis and/or Onchocerciasis	Trachoma	HAT and/or Leishmaniasis
**Middle East**								
Afghanistan	29 million	+	+	+	−	−	+	+
Iran	68 million	5.1 million	1.6 million	0.4 million	−	−	+	+
Iraq	25 million	1.1 million	0.2 million	+	30,000	−	+	+
Syria	18 million	+	+	+	3,878	−	−	+
**Sub-Saharan Africa**								
Angola	13 million	3.2 million	0.4 million	11.4 million	6.1 million	+	+	+
Central African Republic	4 million	0.5 million	0.3 million	1.5 million	0.4 million	+	+	+
Democratic Republic of the Congo	51 million	23.1 million	25.9 million	31.0 million	14.9 million	+	+	+
Somalia	9 million	1.6 million	3.3 million	1.6 million	1.8 million	+	+	+
Sudan	33 million	0.4 million	0.4 million	8.1 million	5.0 million	+	+	+
Zimbabwe	13 million	0.6 million	1.0 million	8.1 million	5.2 million	+	+	+
At-risk sub-Saharan African countries	123 million	29.4 million	31.3 million	61.7 million	33.4 million	+	+	+
**Asia**								
Democratic People's Republic of Korea	22 million	8.0 million	0.2 million	1.3 million	−	−	−	−
Myanmar	49 million	30.2 million	21.3 million	1.3 million	−	>2 million	+	−
At-risk Asian countries	71 million	38.2 million	21.5 million	2.6 million	−	+	+	−
**Latin America & Caribbean**								
Colombia	43 million	6.1 million	15.4 million	3.0 million	−	+	−	+
Cuba	11 million	0.5 million	2.8 million	1.4 million	−	−	−	−
Haiti	8 million	2.6 million	3.8 million	0.8 million	−	+	−	−
Venezuela	25 million	7.4 million	8.7 million	1.6 million	23,674	+	−	+
At-risk countries in the Americas	87 million	16.6 million	30.7 million	6.8 million	0.02 million	+	−	+

+ denotes the presence of a disease but the unavailability of recent prevalence estimates; − denotes no known disease prevalence.

Sources: [15], [19], [20], [22]–[27].

The staggering rates of NTDs that occur largely in problematic countries (in terms of their link with US foreign policy) suggest a vital role for medical intervention against these diseases as an important diplomatic tool. At its simplest, medical diplomacy is “the winning of hearts and minds of people in the Middle East, Asia, Africa, and elsewhere by exporting medical care, expertise, and personnel to help those who need it most” [28],[29]. For example, according to Karen Hughes, former Under Secretary for Public Diplomacy and Public Affairs in the Bush Administration, approximately 87% of people of Bangladesh had a more favorable opinion about the US as a result of a visit of the hospital ship USNS Mercy, a joint initiative of the US Navy and Project Hope [30]. Similar results were noted shortly after the US sent humanitarian relief to Indonesia and elsewhere in the days and weeks following the 2004 Christmas tsunami. As Thompson has argued elsewhere, acts of compassion destroy the rhetoric of terrorists, and the world responds best to America when it provides medical humanitarian relief to the world's war-torn and poorest regions [28],[29].

Through widespread control and elimination of the NTDs, we believe that multiple opportunities exist to expand the concept of medical diplomacy and to make the control and eventually the elimination of NTDs a more visceral and essential element of US foreign policy. As the most common afflictions in the world's areas of conflict and strife, and among the most common bases for diminished agricultural productivity, food insecurity, ignorance, and community destabilization, NTDs represent an obvious target for medical diplomacy. NTD control is also highly cost-effective: in sub-Saharan Africa and elsewhere, control or elimination of several NTDs, including ascariasis, trichuriasis, lymphatic filariasis, trachoma, and onchocerciasis, can be achieved for approximately US$0.50 per person per year, a fraction of the costs for antiretroviral treatment for HIV/AIDS or direct observed therapy of tuberculosis [8],[24]. In practical terms, this means that the entire at-risk populations of war-torn areas and areas of conflict in sub-Saharan Africa listed in Table 1 could be treated for one year at roughly the cost of one or two F/A-18 Hornet fighter jets [31].

We now need to examine mechanisms for embracing NTD control as a critical element of US foreign policy. In addition to providing support for national control programs through public sector funds funneled through the US Agency for International Development and private funds through the Global Network for NTDs [8], a comprehensive policy that includes NTD control could also require assigning specialists in this area to American embassies in low-income and middle-income countries, and cooperative efforts between national health ministries and the US diplomatic corps [18]. These efforts represent fundamental humanitarian assistance, which is consistent with our shared American values of fairness and equity [18],[21].

Medical research for the development of a new generation of drugs, diagnostics, and vaccines for NTDs also affords multiple opportunities for medical diplomacy. Just as the oral polio vaccine was developed as a bilateral US and Soviet medical research program conducted during the height of the Cold War [2]–[4], the US could also now reach out to developing low-income and middle-income countries for joint NTD vaccine development programs. For example, the Americans, the Israelis, and the Iranians are each working independently to study the immunology of human leishmaniasis or to develop leishmaniasis vaccines [32]. Leishmaniasis is one of the most important NTDs in areas of conflict in the Middle East, and was a factor in the Iran–Iraq conflict during the 1980s [33]. The US, Israel, and Iran could work together on a joint initiative to rapidly accelerate the development of a safe and effective leishmaniasis vaccine. Similarly, a vaccine that simultaneously targets hookworm and schistosomiasis, two of the most common NTDs in areas of African conflict, has been launched as a multilateral initiative by the Americans, Brazilians, and Australians [14] and could be extended to the developing nations where both infections are co-endemic.

In addition to the high rates of economic return for the treatment of NTDs through increased agricultural productivity, educational benefits, and other factors (for instance, the economic rate of return for onchocerciasis control has been measured as up to 17% or more [15],[34]), the impact on conflict resolution is potentially enormous. Highly cost-effective NTD control measures need to be fully embraced not only by public health experts and biomedical scientists, but also by the foreign policy community. Doing so would be a sign that the US firmly understands its place in the world and its responsibility to its founding principles and values.

**Box 1. Conflict-Exacerbating Elements of the NTDs**

- Reductions in agricultural productivity
- Abandonment of agricultural lands
- Pivotal role in world's food crisis
- Reductions in education and future wage earning
- Promotion of ignorance
- Adverse child and maternal health
- Community destabilization

## References

[1] Collier P (2007). The bottom billion: Why the poorest countries are failing and what can be done about it.

[2] Hotez PJ (2001). Vaccine diplomacy.. Foreign Policy.

[3] Hotez PJ (2001). Vaccines as instruments of foreign policy. The new vaccines for tropical infectious diseases may have unanticipated uses beyond fighting diseases.. EMBO Rep.

[4] Hotez PJ (2002). Appeasing Wilson's ghost: The expanded role of the new vaccines in international foreign policy..

[5] Schneider M, Moodie M (2002). The destabilizing impacts of HIV/AIDS. Center for Strategic and International Studies 2008.. http://www.kaisernetwork.org/health_cast/uploaded_files/Destabilizing_impacts_of_AIDS.pdf.

[6] Levy B, Sidel V (2008). War and public health.

[7] Utzinger J, Weiss MG (2007). Editorial: Armed conflict, war and public health.. Trop Med Int Health.

[8] Hotez PJ, Molyneux DH, Fenwick A, Kumaresan J, Sachs SE (2007). Control of neglected tropical diseases.. N Engl J Med.

[9] Ramaiah KD, Radhamani MP, John KR, Evans DB, Guyatt H (2000). The impact of lymphatic filariasis on labour inputs in southern India: Results of a multi-site study.. Ann Trop Med Parasitol.

[10] Hotez PJ, Brooker S, Bethony JM, Bottazzi ME, Loukas A (2004). Hookworm infection.. N Engl J Med.

[11] Friedman JF, Kanzaria HK, McGarvey ST (2005). Human schistosomiasis and anemia: The relationship and potential mechanisms.. Trends Parasitol.

[12] Hotez PJ, Molyneux DH, Fenwick A, Ottesen E, Ehrlich Sachs S (2006). Incorporating a rapid-impact package for neglected tropical diseases with programs for HIV/AIDS, tuberculosis, and malaria.. PLoS Med.

[13] Brooker S, Akhwale W, Pullan R, Estambale B, Clarke SE (2007). Epidemiology of *plasmodium-helminth* co-infection in Africa: Population at risk, potential impact on anemia, and prospects for combining control.. Am J Trop Med Hyg.

[14] Hotez PJ, Bethony J, Costa Oliveira S, Brindley PJ, Loukas A (2008). A multivalent anthelminthic vaccine to prevent hookworm and schistosomiasis: A new tool for disease control and sustainable poverty reduction.. Expert Rev Vaccines.

[15] Amazigo U, Noma M, Bump J, Benton B, Liese B, Jamison DT, Feachem RG, Makgoba MW, Bos ER, Bingana FK (2006). Chapter 15: Onchocerciasis.. Disease and mortality in sub-Saharan Africa. 2nd Edition.

[16] Hotez P (2008). Hookworm and poverty.. Ann N Y Acad Sci.

[17] Bleakley H (2007). Disease and development: Evidence from hookworm eradication in the American South.. Q J Econ.

[18] Hotez PJ (2006). The “biblical diseases” and U.S. vaccine diplomacy.. Brown J World Aff.

[19] Molyneux DH (2006). Control of human parasitic diseases: Context and overview.. Adv Parasitol.

[20] Beyrer C, Villar JC, Suwanvanichkij V, Singh S, Baral SD (2007). Neglected diseases, civil conflicts, and the right to health.. Lancet.

[21] Hotez PJ (2008). Forgotten people and forgotten diseases: The neglected tropical diseases and their impact on global health and development.

[22] de Silva NR, Brooker S, Hotez PJ, Montresor A, Engels D (2003). Soil-transmitted helminth infections: Updating the global picture.. Trends Parasitol.

[23] Steinmann P, Keiser J, Bos R, Tanner M, Utzinger J (2006). Schistosomiasis and water resources development: Systematic review, meta-analysis, and estimates of people at risk.. Lancet Infect Dis.

[24] Molyneux DH, Hotez PJ, Fenwick A (2005). “Rapid-impact interventions”: How a policy of integrated control for Africa's neglected tropical diseases could benefit the poor.. PLoS Med.

[25] Polack S, Brooker S, Kuper H, Mariotti S, Mabey D (2005). Mapping the global distribution of trachoma.. Bull World Health Organ.

[26] World Health Organization (2008). Global health atlas.. http://www.who.int/GlobalAtlas/.

[27] Alvar J, Yactayo S, Bern C (2006). Leishmaniasis and poverty.. Trends Parasitol.

[28] Thompson TG (2005). The cure for tyranny. The Boston Globe.. http://www.boston.com/news/globe/editorial_opinion/oped/articles/2005/10/24/the_cure_for_tyranny/.

[29] Iglehart J (2008). Advocating for medical diplomacy: A conversation with Tommy G. Thompson. Project HOPE–The People-to-People Health Foundation.. http://content.healthaffairs.org/cgi/content/full/hlthaff.w4.262v1/DC1.

[30] Hughes K (2006). Medical diplomacy. Why Mercy Matters Conference.. http://www.state.gov/r/us/77295.htm.

[31] Angels Blue (2008). Frequently asked questions.. http://www.blueangels.navy.mil/index.htm.

[32] Hotez PJ, Ferris MT (2006). The antipoverty vaccines.. Vaccine.

[33] Hosseini SM, Hatam GR, Ardehali S (2005). Characterization of leishmania isolated from unhealed lesions caused by leishmanization.. East Mediterr Health J.

[34] Kim A, Bendon B (1995). Cost-benefit analysis of the onchocerciasis control program (OCP).

